# Predicting central lymph node metastasis in papillary thyroid microcarcinoma: a study of ultrasound and clinical features

**DOI:** 10.3389/fendo.2026.1752405

**Published:** 2026-04-10

**Authors:** Xiongqiang Peng, Jianxin Zhang, Yiyang Lin, Ruizhuo Li

**Affiliations:** 1 Department of Medical Ultrasonics, Guangdong Provincial Hospital of Traditional Chinese Medicine, Guangzhou, China; 2 The Second Affiliated Hospital of Guangzhou University of Chinese Medicine, Guangzhou, China; 3 School of Medicine, Guangzhou University of Chinese Medicine, Guangzhou, China

**Keywords:** central lymph node metastasis (CLNM), papillary thyroid microcarcinoma (PTMC), prediction model, radiomics, ultrasound features

## Abstract

**Background:**

Papillary thyroid microcarcinoma (PTMC) generally has a favorable prognosis. Early central lymph node metastasis (CLNM) can significantly impact treatment strategy and prognosis. However, CLNM lacks typical ultrasound features. Accurate preoperative prediction of CLNM remains challenging. This study aims to develop and validate a high-accuracy tool for preoperatively assessing the risk of lymph node metastasis in PTMC patients.

**Methods:**

We retrospectively analyzed clinical and ultrasound data from 534 PTMC patients who underwent initial thyroidectomy with central lymph node dissection. Patients were randomly divided into training (n=373) and validation (n=161) cohorts. We calculated high-throughput radiomics features, including tumor size, tumor shape, margin, capsular contact, microcalcifications, and peritumoral echogenicity features. A combined feature selection strategy was then used to identify features with the greatest discriminatory power for lymph node status. A Logistic Regression machine classifier was employed to build and validate the prediction model. Additionally, ultrasound ACR TI-RADS and clinical variables were evaluated. Univariate and multivariate logistic regression was used to identify independent predictors, which were further incorporated into a nomogram model. The area under the operating characteristic curves (AUCs) was used to draw comparisons between different models and the decision curve analysis was conducted to assess their clinical utility.

**Results:**

In the clinical model based solely on clinical and conventional ultrasound features, multivariate analysis identified five independent predictors of CLNM: age <46.5 years, male sex, capsular contact ≥50%, peritumoral hyperechogenicity and heterogeneous echotexture (AUC: 0.857 in the training set and 0.840 in the validation set). By further integrating a radiomics score with all univariately significant clinical variables, a combined clinical-radiomics nomogram was developed. In this combined model, age, transverse diameter of tumor, capsular contact, peritumoral echo changes, and the radiomics score were identified as independent predictors. The combined model achieved an improved AUC of 0.900 in the validation set, demonstrating superior predictive performance and higher clinical net benefit than the clinical model alone.

**Conclusion:**

The proposed clinical-radiomics nomogram, which incorporates conventional ultrasound features and radiomics signatures, outperforms the standalone clinical model in predicting CLNM. This non-invasive approach provides superior pre-operative risk assessment in optimizing treatment strategies for PTMC patients.

## Introduction

Recent advances in computed tomography, deep learning, and radiomics have significantly improved the prediction of lymph node metastasis in thyroid cancer (1, 2). Concurrently, the detection of papillary thyroid microcarcinoma (PTMC, ≤1 cm) has substantially increased (3), raising prominent concerns regarding over-diagnosis and the overtreatment of tumors (1), which have limited effectiveness (4, 5). This trend is particularly evident in China, where the incidence of thyroid cancer surged by 289.6% from 1990 to 2019 (6), underscoring an urgent need for enhanced prevention, control, and more conservative management strategies (7, 8). Directly applying predictive models developed for larger tumors to PTMC is hindered by its small size and often atypical and limited imaging features (7, 9, 10), resulting in a persistent lack of high-accuracy tools for preoperative risk assessment. Furthermore, central lymph node metastasis (CLNM) lacked the typical ultrasound features of microcalcifications (1), such as partially cystic appearance, increased vascularization (5). Moreover, there are anatomic areas of the central region that are not well visualized by ultrasound, such as the posterior tracheal area, posterior esophageal area, posterior pharyngeal area, and mediastinal area (11). In clinical practice, ultrasound sensitivity for central nodes is as low as 33% (12). The goal of the predictive nomogram is to bridge the gap between preoperative occult status and postoperative confirmed pathology.

As active surveillance (AS) becomes a standard for low-risk PTMC, the key challenge shifts to its safe implementation (8). This necessitates reliably excluding patients with occult central lymph node metastasis (CLNM)—a clinically significant finding with a high prevalence (up to 60.9%) that is linked to higher recurrence (13, 14). Accurate preoperative CLNM prediction is therefore paramount, enabling tailored surgical approaches that balance cure with quality of life (15).

Prior CLNM prediction models for PTMC, often focused on limited features, show restricted discriminatory power (16, 17) and have either relied on clinical variables (12, 18) or merely focused on the thyroid ultrasound images (19). A unique strength of this research is the systematic evaluation of both transverse and longitudinal ultrasound views. Traditional radiomics often focuses on the single plane with the maximum tumor diameter, but PTMC growth is frequently asymmetric (20). By capturing high-dimensional data from two orthogonal planes, the model identifies previously neglected morphological patterns. This innovative approach enables our model to quantify individual CLNM risk with unprecedented precision, strategically guiding patient management to ensure active surveillance is applied to appropriate candidates while reserving surgery for those who will truly benefit.

## Materials and methods

**Figure 1:**
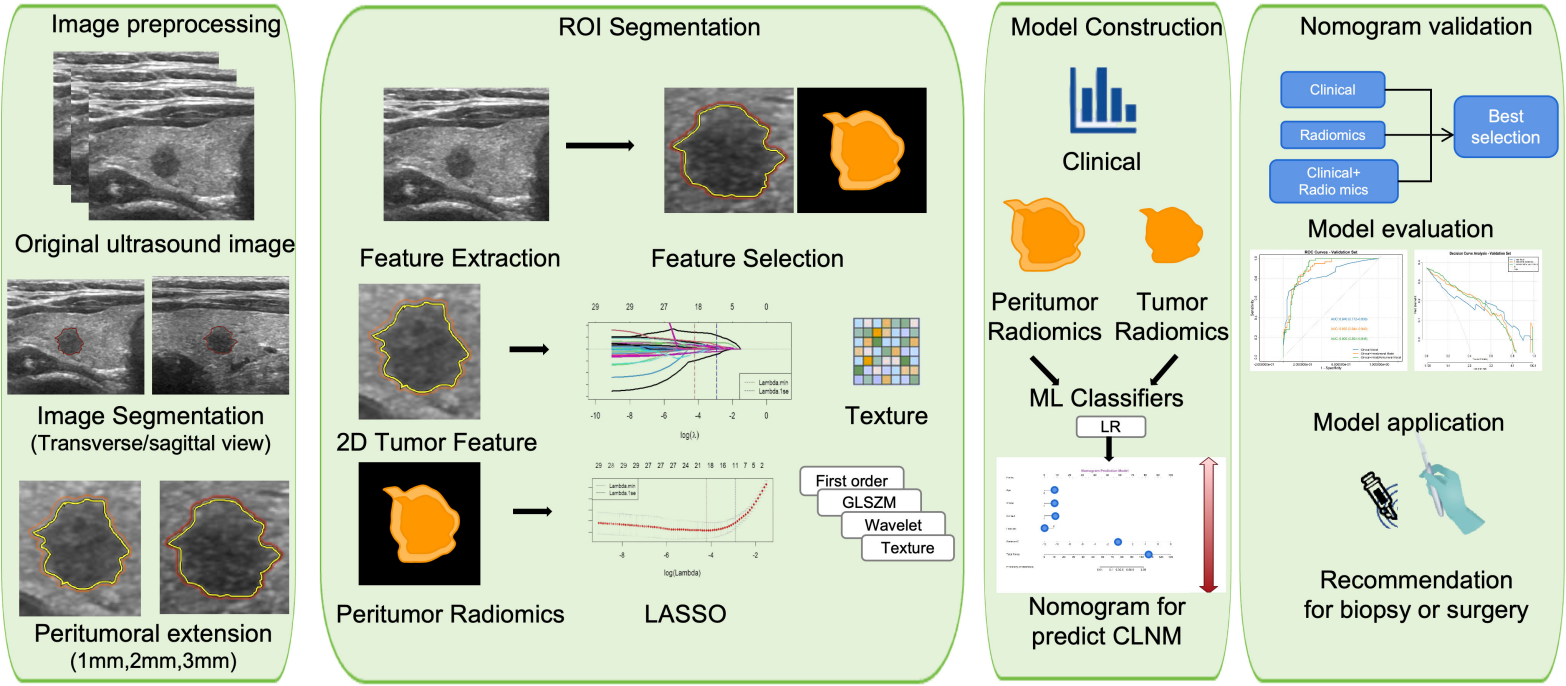
Workflow of the lymph node metastasis (LNM) prediction system. (I) Manual segmentation of thyroid ultrasound images in transverse and sagittal views, including peritumoral extension. (II) Extraction of radiomics features from the region of interest (ROI), including texture, first-order, GLSZM, and wavelet features. (III) Feature selection using methods such as LASSO. (IV) Model construction using machine learning classifiers (LR) and integration into a nomogram combined with clinical factors for predicting central lymph node metastasis (CLNM). (V) Model validation and evaluation, supporting clinical recommendations for biopsy or surgery.

In this study, we proposed both radiomics evaluation and traditional nomogram construction based on preoperative US thyroid images to predict CLNM noninvasively in patients with PTMC. The intra-tumor and peritumor radiomics evaluation including features from three consecutive annular expansions beyond the tumor contour: 1 mm, 2 mm, and 3 mm. This multi-shell approach was designed to empirically determine the spatial scale at which peritumoral radiomic features provide the most discriminative power. Following feature selection, a machine learning method was applied. The workflow of our study is shown in **Figure 1**.

### Study population

A retrospective analysis was conducted on 593 patients with pathologically confirmed papillary thyroid microcarcinoma (PTMC) who underwent initial surgical treatment at Guangdong Provincial Hospital of Traditional Chinese Medicine between February 2022 and April 2023. Inclusion criteria were: (1) initial thyroidectomy with cervical lymph node dissection and pathological PTMC diagnosis; (2) preoperative ultrasound with transverse or sagittal views and high-quality images; (3) complete clinical and laboratory data; and (4) no history of anticancer therapy before surgery. Exclusion criteria included: (1) non-PTMC pathology or no lymph node dissection; (2) previous thyroid cancer surgery or related treatment; (3) unclear ultrasound images or incomplete nodule visualization; (4) incomplete clinical data; and (5) age under 18 years, pregnancy, lactation, or severe cardiopulmonary, cerebrovascular, or psychiatric disorders. Based on these criteria, 59 cases were excluded for: inadequate image quality (n=15), no lymph node dissection (n=9), preoperative biopsy (n=7), postoperative recurrence requiring reoperation (n=17), absence of preoperative ultrasound (n=10), and co-occurrence of other tumors (n=1).

A total of 534 patients with pathologically confirmed central lymph node metastasis were retrospectively enrolled in this study (**Table 1**; **Supplementary Figure 1**). Using a computer-generated random function, the entire cohort was partitioned into a training set and a validation set at a ratio of 7:3. This resulted in 373 cases assigned to the training set and 161 cases to the validation set. Based on the postoperative pathological confirmation of central lymph node metastasis status, the patients were further categorized into a metastasis group and a non-metastasis group. The training set comprised 140 patients with metastasis and 233 without, while the validation set comprised 60 patients with metastasis and 101 without. Extra validation cohort came from the affiliated hospital of Zunyi Medical University which comprised 50 patients with 15 metastasis and 35 without. This study was approved by the institutional ethics committee (Approval No. BE2024-303-01). As a retrospective study, the requirement for informed consent was waived.

**Table 1:** Baseline characteristics of PTMC patients in training and validation cohorts.

Risk factor	Training cohorts	Validation cohorts	χ²/Z value	*P* value
Clinical Characteristics				
Gender (Male vs Female)	83 (22.25%)	40 (24.84%)	0.426	0.514
Age (years)	43.00 (35.00,52.00)	45.00 (37.00,53.50)	-1.214	0.225
T3 (IU/mL)	1.69 (1.52,1.91)	1.71 (1.52,1.90)	-0.287	0.774
T4 (IU/mL)	106.24 (92.43,119.98)	105.50 (90.53,120.62)	-0.026	0.980
FT3 (IU/mL)	5.16 (4.77,5.65)	5.20 (4.82,5.61)	-0.253	0.800
FT4 (IU/mL)	16.01 (14.49,17.77)	16.00 (14.56,17.40)	-0.029	0.977
ATPO (IU/mL)	33.00 (28.00,53.96)	34.40 (28.00,107.658)	-0.515	0.607
TSH (mIU/L)	1.33 (0.84,1.98)	1.48 (0.96,2.04)	-1.535	0.125
Blood Creatinine (μmol/L)	58.0 (51.00,71.00)	58.00 (52.00,70.50)	-0.341	0.733
Blood Glucose (mmol/L)	5.04 (4.69,5.57)	5.06 (4.70,5.67)	-0.474	0.636
Lesion Characteristics				
Anteroposterior Diameter (mm)	6.00 (4.50,8.00)	6.00 (5.00,8.00)	-0.279	0.781
Craniocaudal Diameter (mm)	6.00 (4.00,7.00)	6.00 (5.00,7.00)	-0.232	0.817
Transverse Diameter (mm)	6.00 (4.00,7.00)	6.00 (4.50,7.00)	-0.302	0.763
Maximum Diameter (mm)	7.00 (5.00,9.00)	7.00 (5.00,8.00)	-0.100	0.920
Sum of Diameters (mm)	7.00 (5.00,9.00)	7.00 (5.00,9.00)	-0.290	0.772
Multiplicity	23 (6.17%)	12 (7.45%)	0.304	0.581
Location (Isthmus vs Lobe)	31 (8.31%)	12 (7.45%)	0.112	0.738
Shape (Irregular vs Regular)	337 (90.35%)	148 (91.93%)	0.336	0.562
Margin (Ill-defined vs Well-defined)	330 (88.47%)	145 (90.06%)	0.289	0.591
**Aspect** Ratio (Taller-than-wide vs Wider-than-tall)	274 (73.46%)	116 (72.05%)	0.113	0.736
Degree of Capsular Contact (≥50% vs <50%)	154 (41.29%)	66 (40.99%)	-0.063	0.950
Homogeneity (Heterogeneous vs Homogeneous)	251 (67.29%)	114 (70.81%)	0.642	0.423
Peritumoral Echogenic Changes (Present vs Absent)	96 (25.74%)	45 (27.95%)	0.283	0.594
Posterior acoustic pattern (Attenuated, vs Enhanced, mixing, or shadowing)	38 (10.19%)	14 (8.70%)	0.285	0.594
Microcalcifications (Present vs Absent)	189 (50.67%)	83 (51.55%)	-0.187	0.852
ACR TI-RADS Score (High vs Low)	101 (72.14%)	151 (64.81%)	-1.463	0.143

### Image acquisition and ultrasound feature analysis

Ultrasound images were acquired using multiple devices, including GE vivid E9, Logiq E20, Logiq E9 (General Electric Co.); Philips EPIQ7, EPIQ5 (Philips Medical Systems); and Toshiba Aplio 500 (Toshiba), from two branches of the Guangdong Provincial Hospital of Traditional Chinese Medicine. Extra validation images were all acquired using GE Logiq E20. All examinations were performed using high-frequency linear array transducers (ranging from 5 to 14 MHz). Static B-mode images were exported in standard Digital Imaging and Communications in Medicine (DICOM) format prior to analysis. Imaging protocols and operational procedures were standardized across all machines and sites to ensure consistency.

Two experienced radiologists, blinded to all clinical and pathological data, independently assessed the following ultrasound features based on predefined binary classification criteria (**Table 2** for the binary analysis) including location (isthmus or lobe), shape (irregular or regular), margin (ill-defined or well-defined), aspect ratio (taller-than-wide or wider-than-tall, degree of Capsular Contact (≥50% or <50%), homogeneity (heterogeneous or homogeneous), peritumoral echogenic changes (present or absent), posterior acoustic pattern (attenuated vs. enhanced/mixed/shadowing) and microcalcifications (present or absent). To ensure objectivity, the radiologists were provided with no demographic or clinical information during the assessment. Any discrepancies in their independent assessments were resolved through consensus discussion.

**Table 2:** Logistic regression analysis of single factor CLNM in PTMC patients.

Risk factor	CLNM (+) (n=140)	CLNM (-) (n=233)	χ²/Z value	*P* value
Clinical Characteristics				
Gender (Male vs Female)	47 (33.57%)	36 (15.45%)	16.599	<0.001
Age (years)	39.00 (33.25,47.75)	45.00 (37.00,53.50)	-3.708	<0.001
T3	1.68 (1.52,1.91)	1.71 (1.52,1.90)	-0.590	0.555
T4	102.21 (91.63,118.30)	106.83 (93.01,121.98)	-1.142	0.253
FT3	5.17 (4.78,5.65)	5.15 (4.76,5.66)	-0.183	0.854
FT4	16.11 (14.47,17.86)	15.88 (14.52,17.67)	-0.602	0.547
ATPO (IU/mL)	30.74 (28.00,46.80)	34.50 (28.00,57.08)	-1.927	0.054
TSH (mIU/L)	1.27 (0.86,1.87)	1.39 (0.82,2.14)	-0.763	0.446
Blood Creatinine (μmol/L)	61.5 (54.00,75.00)	57.00 (50.00,69.00)	-3.229	0.001
Blood Glucose (mmol/L)	4.97 (4.69,5.64)	5.06 (4.76,5.56)	-0.095	0.924
Lesion Characteristics				
Anteroposterior Diameter (mm)	7.00 (5.00,9.00)	6.00 (4.00,8.00)	-3.085	0.002
Craniocaudal Diameter (mm)	6.00 (5.00,8.00)	6.00 (4.00,7.00)	-3.307	<0.001
Transverse Diameter (mm)	6.00 (5.00,8.00)	5.00 (4.00,7.00)	-4.012	<0.001
Maximum Diameter (mm)	7.50 (6.00,9.00)	6.00 (5.00,8.00)	-3.518	<0.001
Sum of Diameters (mm)	8.00 (6.00,10.00)	6.00 (5.00,9.00)	-3.861	<0.001
Multiplicity	12 (8.57%)	16 (6.87%)	-1.495	0.135
Location (Isthmus vs Lobe)	15 (10.71%)	11 (4.72%)	1.699	0.192
Shape (Irregular vs Regular)	134 (95.71%)	203 (87.12%)	7.400	0.007
Margin (Ill-defined vs Well-defined)	130 (92.86%)	200 (85.84%)	4.226	0.040
**Aspect** Ratio (Taller-than-wide vs Wider-than-tall)	105 (75.00%)	169 (72.53%)	0.273	0.601
Degree of Capsular Contact (≥50% vs <50%)	95 (67.86%)	59 (25.32%)	-8.068	<0.001
Homogeneity (Heterogeneous vs Homogeneous)	115 (82.14%)	136 (58.37%)	22.457	<0.001
Peritumoral Echogenic Changes (Present vs Absent)	74 (52.86%)	22 (9.44%)	86.243	<0.001
Posterior acoustic pattern (Attenuated, vs Enhanced, mixing, or shadowing)	14 (10.00%)	24 (10.30%)	0.009	0.926
Microcalcifications (Present vs Absent)	86 (61.43%)	103 (44.21%)	-3.217	0.001
ACR TI-RADS Score (High vs Low)	101 (72.14%)	151 (64.81%)	-1.463	0.143

### Radiomics feature extraction

Radiomics feature extraction was performed on the largest cross-sectional and longitudinal images of each lesion using the PyRadiomics library within the DARWIN Medical AI Platform (Yizhun Intelligent, Beijing, China), version 3.3.7. Initially, a total of 1125 features were extracted from each of the two views per lesion, yielding a potential 2250 features per region (intratumoral, peritumoral 1mm, 2mm, and 3mm). To ensure feature robustness, 51 features containing zero values were excluded, as these represent mathematically unstable or uninformative matrices inherent to the small pixel arrays of microcarcinomas. Consequently, a refined and consistent set of 2148 radiomics features was ultimately included for the analysis of each respective region (intratumoral and each peritumoral expansion). These features comprehensively captured the tumor phenotype, encompassing: First-order statistics which describe the distribution of voxel intensities (e.g., mean, median), 2D shape-based features which quantify the geometric properties of the lesion and Second-order and higher-order texture features which characterize the spatial relationships of voxel intensities, derived from Gray Level Co-occurrence Matrix (GLCM), Gray Level Run Length Matrix (GLRLM), Gray Level Size Zone Matrix (GLSZM), Gray Level Dependence Matrix (GLDM), and Neighboring Gray Tone Difference Matrix (NGTDM).

### Radiomics feature selection and model construction

The feature selection pipeline was rigorously applied to the training set to ensure robustness and prevent overfitting. The process involved the following steps:

Feature Reproducibility Assessment: To evaluate the consistency of feature extraction, a random subset of 50 cases was selected. Two radiologists independently delineated the ROIs to assess inter-observer agreement, and one radiologist repeated the segmentation after one month to assess intra-observer agreement. Small ROIs in PTMC are highly sensitive to single-pixel shifts in manual segmentation, which can lower the ICC of complex texture features (21).The Intraclass Correlation Coefficient (ICC) was calculated, and only features with high reproducibility (ICC > 0.71) were retained for subsequent analysis. Finally, 1964 out of 2148 radiomics features were selected to construct the model.
Data Preprocessing and Harmonization: To mitigate batch effects from different ultrasound devices—including the GE Vivid E9, Logiq E9, Philips EPIQ 7, and Toshiba Aplio 500—we implemented a rigorous harmonization and standardization pipeline. The ComBat harmonization method (22), which utilizes an Empirical Bayes framework to adjust for mean and variance shifts associated with different scanner models while preserving essential biological variance, was employed as the primary correction tool (23). The effectiveness of ComBat harmonization in removing scanner-specific batch effects is visualized in **Supplementary Figure 6** via PCA clustering. These fitted parameters were subsequently applied to transform the independent validation set. Following harmonization, each feature underwent Z-score normalization to standardize the distribution to a mean of 0 and a standard deviation of 1 across all samples.
Dimensionality Reduction: Features with near-zero variance were first removed. To address multicollinearity, highly correlated features (correlation coefficient > |0.9|) were filtered out, retaining the one with higher clinical relevance. Finally, the Least Absolute Shrinkage and Selection Operator (LASSO) regression algorithm with 10-fold cross-validation was employed once on the entire training set for feature selection and further dimensionality reduction. The optimal penalty parameter (lambda) was determined based on the minimum binomial deviance criterion (lambda.min), resulting in a sparse set of the most predictive non-redundant features for final model construction. Our final combined model included 12 predictors in total (4 clinical and 8 radiomics features). This yields an Event-to-Variable Ratio of 10.77 (140/12), which exceeds the widely accepted threshold of 10 events per variable required to ensure model stability and minimize the risk of overfitting.

### Statistical analysis and model development

Univariate and multivariate logistic regression analyses were performed to identify independent predictors of CLNM. The discriminative performance of the radiomics models, constructed from peritumoral features extracted at varying peritumoral distances, was evaluated and compared in the test set. To statistically compare models evaluating different peritumoral distances, DeLong’s test for correlated ROC curves was employed. For the visualization of all models, a nomogram constructed using significant variables. Logistic regression (LR) classifiers were used to train radiomic models, and their performance was evaluated based on the results from the testing cohort. The radiomic model was evaluated using a set of performance metrics, including accuracy (ACC), precision, recall, F1 score, and receiver operating characteristic (ROC) curve analysis and DCA, in both the training and testing cohorts. Statistical analyses were conducted using SPSS 26.0 and R software.

## Results

### Univariable and multivariable analysis for CLNM

Univariate analysis revealed significant associations between CLNM and age, sex, tumor size, shape, margin, capsular contact, echogenicity, microcalcifications, and peritumoral echogenicity (**Table 2**). Multivariate analysis identified five independent predictors: age <46.5 years, male sex, heterogeneous echotexture, capsular contact ≥50%, and peritumoral hyperechogenicity (**Table 3**).

**Table 3:** Multivariable logistic regression analysis of CLNM in PTMC patients.

Risk factor	β coefficient	Std. error	*Wald*	*P*	*OR*	95%*CI* for OR
Age	-0.052	0.014	13.989	<0.001	0.950	0.924~0.976
Gender (Male vs Female)	1.224	0.431	8.064	0.005	3.400	1.461~7.914
Creatinine	-0.002	0.010	0.033	0.856	0.998	0.978~1.019
Anteroposterior Diameter	-0.374	0.228	2.707	0.100	0.688	0.440~1.074
Craniocaudal Diameter	-0.150	0.138	1.190	0.275	0.860	0.657~1.127
Transverse Diameter	0.188	0.158	1.419	0.234	1.207	0.886~1.644
Maximum Diameter	0.174	0.301	0.334	0.563	1.190	0.660~2.147
Sum of Diameters	0.089	0.053	2.830	0.093	1.093	0.985~1.213
Shape (Irregular vs Regular)	0.926	0.702	1.738	0.187	2.524	0.637~9.996
Margin (Ill-defined vs Well-defined)	0.125	0.570	0.048	0.827	1.133	0.371~3.459
Homogeneity (Heterogeneous vs Homogeneous)	0.809	0.397	4.156	0.041	2.245	1.032~4.885
Peritumoral Echogenic Changes (Present vs Absent)	2.104	0.328	41.037	<0.001	8.202	4.308~15.615
Microcalcifications (Present vs Absent)	-0.025	0.329	0.006	0.938	0.975	0.512~1.856
Degree of Capsular Contact (≥50% vs <50%)	1.624	0.309	27.596	<0.001	5.074	2.768~9.300
Constant	-0.994	1.054	0.891	0.345	0.370	

Multivariable logistic regression analysis, incorporating all significant univariable predictors, identified five independent risk factors for CLNM (+).

In the training cohort, univariable analysis identified multiple factors significantly associated with CLNM (all p < 0.05), including younger age, male sex, larger tumor dimensions, irregular shape, ill-defined margins, capsular contact ≥50%, heterogeneous echotexture, microcalcifications, and peritumoral hyperechogenicity (**Table 2**). Among these, peritumoral hyperechogenicity showed the strongest association (χ² = 86.243, p < 0.001).

### Rationale for peritumoral region selection and model interpretation

The analysis of the peritumoral microenvironment is critical in oncology, as pathological processes such as microinvasion, desmoplastic reaction, and inflammatory changes often initiate and propagate in the tissue immediately surrounding the tumor bulk. To systematically investigate this region and identify the optimal field of view for capturing peritumoral heterogeneity, we defined and extracted features from three consecutive annular expansions beyond the tumor contour: 1 mm, 2 mm, and 3 mm. This multi-shell approach was designed to empirically determine the spatial scale at which peritumoral radiomic features provide the most discriminative power. Subsequent statistical comparison using DeLong’s test revealed that models based on the 2 mm and 3 mm peritumoral regions demonstrated significantly higher predictive performance (AUCs of 0.949 and 0.945, respectively) compared to the 1 mm model (AUC of 0.885), with statistically significant differences (p < 0.001). Since no significant difference was observed between the 2 mm and 3 mm models (p = 0.541), the 2 mm region was selected for subsequent model construction to maximize information yield while minimizing the potential inclusion of irrelevant distant parenchyma.

**Figure 2:**
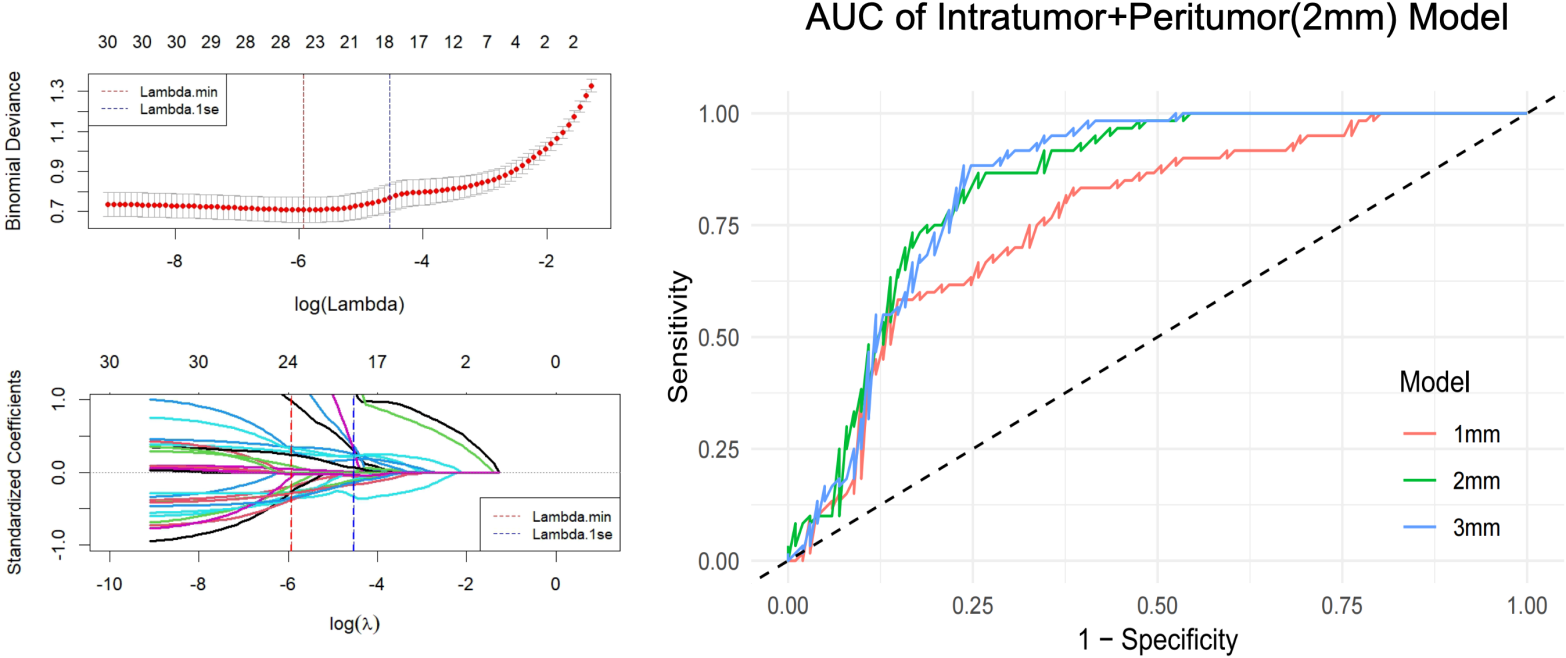
Radiomics feature selection and CLNM prediction model. LASSO regression was employed to select the most predictive features from high-dimensional radiomic data, integrating both intratumoral and peritumoral (2-mm) regions. This combined model captures tumor-intrinsic and microenvironmental alterations, enabling improved preoperative risk stratification for central lymph node metastasis in papillary thyroid microcarcinoma. The combined model improves CLNM risk stratification in papillary thyroid microcarcinoma.

To address the high-dimensional nature of radiomics data and prevent model overfitting, we employed LASSO regression for radiomics feature selection, which effectively identified the most predictive features while eliminating redundant variables. The final radiomics signature integrated both intratumoral and peritumoral (2-mm) features, creating a comprehensive biomarker that captures both tumor-intrinsic characteristics and microenvironmental alterations (**Figure 2**). Among the selected features, lower sphericity values (OR = 0.21-0.33, p<0.001) in both cross-sectional and longitudinal planes emerged as strong protective factors, quantitatively confirming that irregular tumor shape serves as a key indicator of invasive potential. Concurrently, elevated intratumoral heterogeneity (OR = 1.58, p=0.026) reflected by first-order statistics further supported the association between internal architectural disorganization and metastatic behavior. Most notably, peritumoral texture features, particularly dependence entropy (OR = 2.28, p=0.0004) and run entropy (OR = 1.74, p=0.032) demonstrated the highest predictive values, suggesting that microstructural disorganization in the tumor microenvironment provides critical information about early invasive processes (**Supplementary Figure 4**). This combined approach of analyzing both tumor morphology and peritumoral texture patterns provides a more complete assessment of CLNM risk than either domain alone, offering valuable insights for preoperative stratification and personalized surgical planning in PTMC patients.

### Final nomogram construction and performance

**Figure 3:**
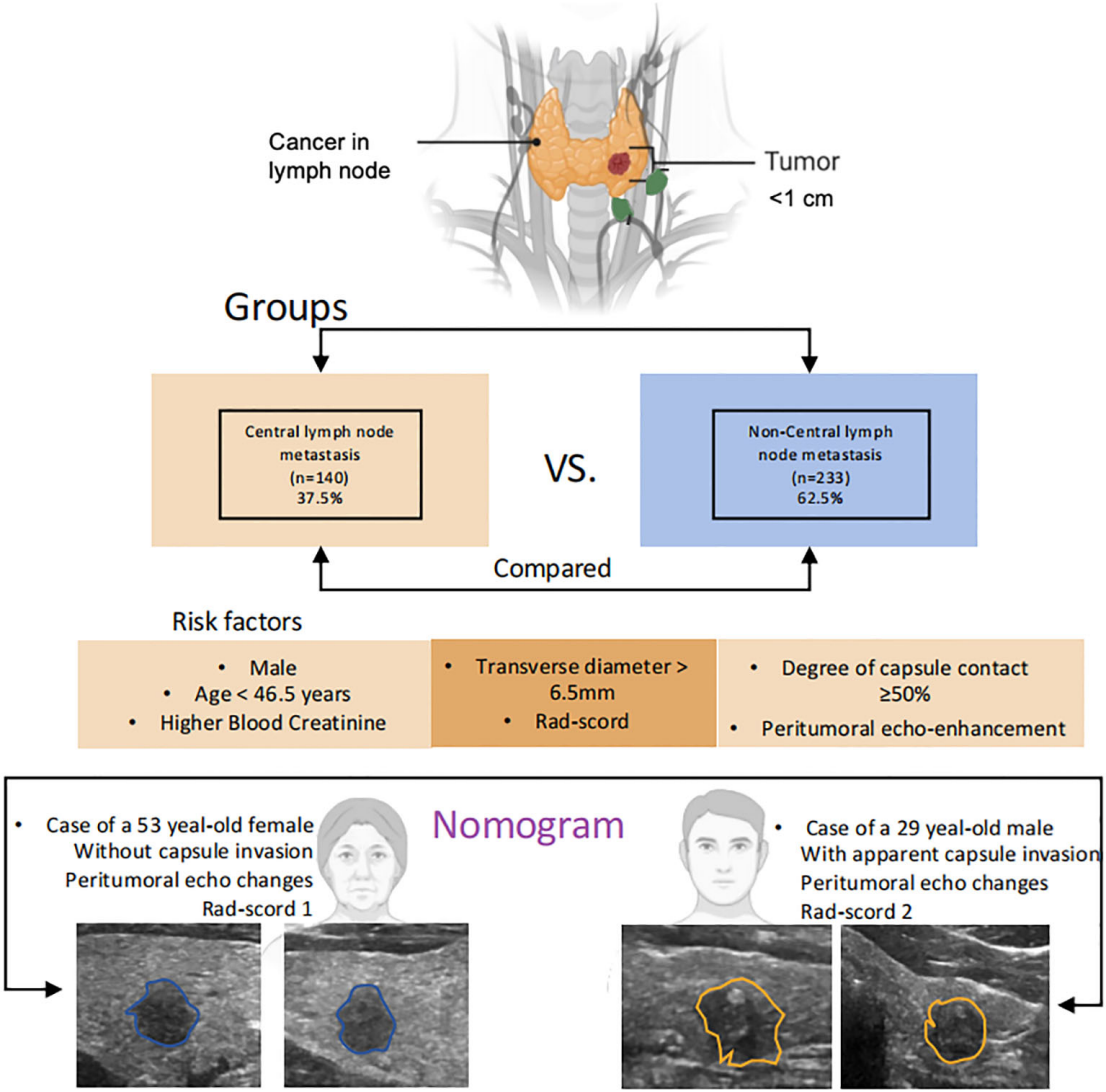
Nomogram model incorporating the mixed risk factors with radiomics and clinical features. Important clinical variables selected by multivariable logistic regression analysis of central lymph node metastasis (CLNM) in papillary thyroid microcarcinoma patients. Two representative cases are present in the visual nomogram: a 29-year-old male patient with an irregular thyroid tumor exhibiting apparent capsule invasion demonstrated an extremely high risk of recurrence of CLNM while a 53-year-old female patient with relative lower risk of CLNM.

To facilitate the intuitive application of our predictive model, we developed a comprehensive nomogram that integrates significant risk factors derived from clinical assessments and radiomics analysis (**Figure 3**). The nomogram visually synthesizes five independent predictors identified through our analysis: age, transverse diameter, degree of capsule contact, peritumoral echo changes, and the Rad-score. Notably, while qualitative heterogeneous echotexture was an independent clinical predictor (**Table 2**), it was substituted in the final nomogram by the Rad-score, which objectively and more precisely quantifies.

**Figure 4:**
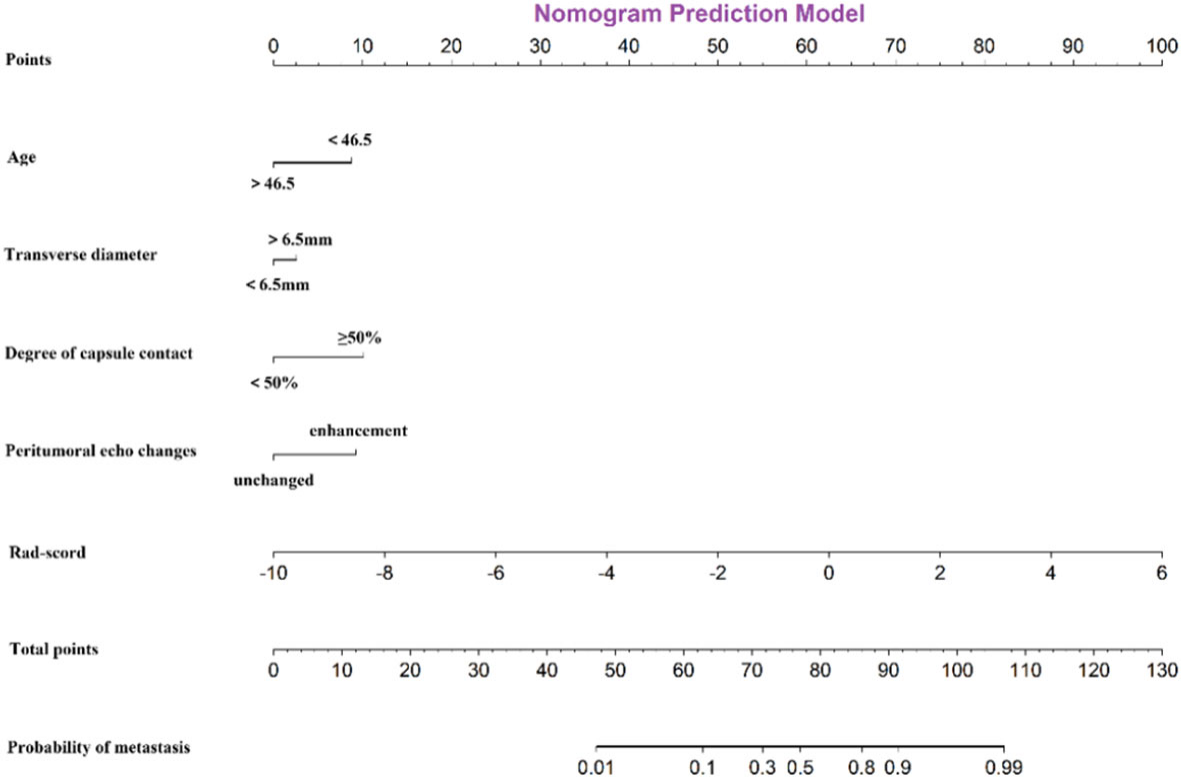
Details and visualization of nomogram. The nomogram integrates five independent predictors identified through multivariate analysis: age, transverse diameter of the tumor, degree of capsule contact, peritumoral echo-enhancement, and the radiomics score (Rad-score). Each variable is assigned points on the top scale. The total points, derived from the sum of individual scores, are projected downward to estimate the individualized probability of CLNM.

To illustrate the clinical application of this nomogram (**Figure 4**), consider a representative case: a young male patient presenting with an irregular thyroid tumor exhibiting apparent capsule invasion and a Rad-score of 2. According to the nomogram scoring system: Age <46.5 years (40 points), transverse diameter >6.5mm (30 points), degree of capsule contact ≥50% (20 points), enhancement of peritumoral echo changes (30 points), Rad-score of 2 (25 points). The cumulative total score of 145 points corresponds to a predicted CLNM risk exceeding 90%, indicating an extremely high probability of metastasis. This quantitative assessment provides clinicians with an evidence-based foundation for recommending aggressive therapeutic interventions, including prophylactic central neck dissection.

To enhance the interpretability of our model, we employed SHAP (SHapley Additive exPlanations) analysis, which quantitatively assesses the contribution of each feature to the model’s predictions. The SHAP results demonstrated that the Rad-score was the most influential predictor in both training and validation sets, with the highest mean |SHAP value| (**Supplementary Figure 2**). This was followed by the degree of capsule contact and age, confirming the robust contribution of the radiomics signature to the model’s decision-making process. The SHAP dependence plot further revealed a nonlinear relationship between Rad-score values and their impact on CLNM risk prediction, providing additional insights into how different ranges of the radiomics signature influence the model’s output.

The Rad-score (Radiomics Score) serves as a quantitative indicator that comprehensively evaluates tumor imaging characteristics through the following formula: Rad-score = β₀ + β_1_f&_1_ + β_2_f_2_ + … + β_n_f_n_, where β₀ represents the intercept, and β_1_…β_n_ denote the weighted coefficients for the selected radiomics features f_1_…f_n_. The detailed calculation formulas for each nomogram’s Rad-score are provided in **Supplementary Materials**.

Specifically, by mapping the SHAP dependence analysis to pre-defined risk tiers, we established clinically actionable Rad-score thresholds: Low Risk (Rad-score < -0.5), where SHAP values are negative and suppress predicted risk; Intermediate Risk (Rad-score -0.5 to 3.5), where SHAP values transition to positive and promote risk; and High Risk (Rad-score > 3.5), where the SHAP contribution reaches a plateau. These thresholds, rounded for clinical convenience, allow for the direct translation of the radiomics signature into surgical decision-making.

To ensure the robustness of our model, we specifically addressed potential multicollinearity among predictors. Variance Inflation Factor (VIF) analysis confirmed the absence of significant multicollinearity, with all values remaining within acceptable limits (VIF<5). This was further validated through correlation heatmap visualization (**Supplementary Figure 3**), which demonstrated no strong correlations between the selected clinical, ultrasound, and radiomics features, confirming that each variable contributes unique predictive information to the model.

This visual predictive nomogram tool allows for individualized assessment of central lymph node metastasis (CLNM) risk in patients with papillary thyroid microcarcinoma. Each predictor is assigned a specific score on the points scale according to its value. The sum of these individual scores yields a total points value, which can be projected to the bottom probability scale to estimate the personalized probability of CLNM. The nomogram demonstrates that higher total scores correspond to increased metastatic risk, with the probability scale ranging from 0.01 to 0.99.

**Figure 5:**
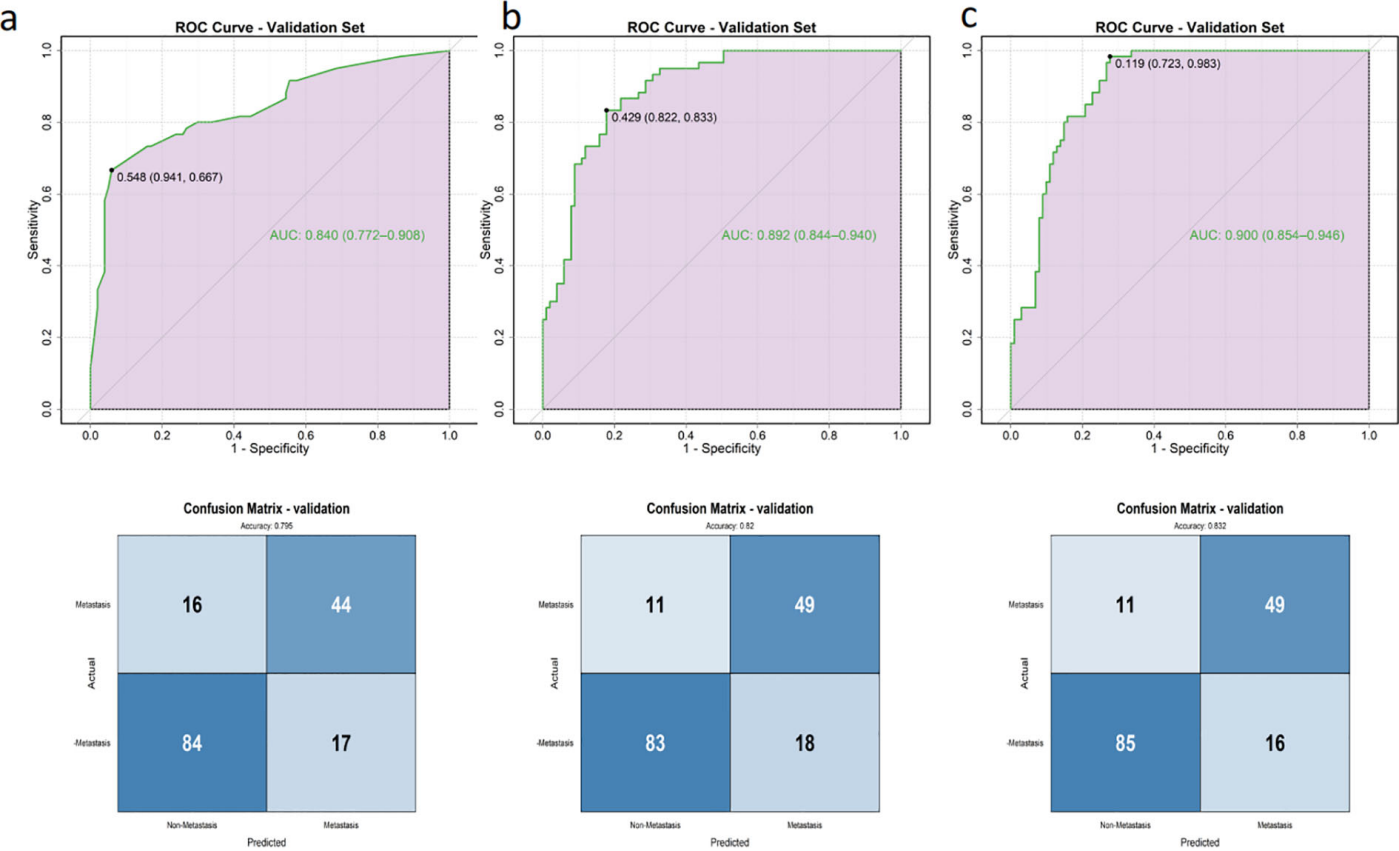
Receiver operating characteristic (ROC) curves of nomograms in the internal validation dataset and associated confusion matrix for the clinical model **(a)**, the clinical + intratumor model **(b)** and the clinical+intra&peritumor model **(c)**.

This integrated predictive tool effectively translates complex clinical and radiomic data into an accessible scoring system, enabling rapid preoperative risk stratification and supporting personalized surgical decision-making for PTMC patients. The clinical model’s ROC curve analysis demonstrated that the age, peritumoral echo changes and degree of capsule contact exhibited a good predictive accuracy for CLNM in the overall population (AUC = 0.840, 95% CI: 0.772–0.908) (**Figure 5a**), and the age optimal cut-off value was determined to 46.5, and the transverse diameter cut-off value was 6.5mm derived from the Youden Index.

The other nomograms also demonstrated excellent discriminative abilities. In the clinical intratumor validation set, the area under the curve (AUC) was 0.892 (95% CI: 0.844–0.940) (**Figure 5b**). The clinical+intra&peritumor model maintained strong performance in the validation set, with an AUC of 0.900 (95% CI: 0.854–0.946) (**Figure 5c**). Additionally, in the external validation (n=50), the model demonstrated robust performance with an AUC of 0.943. Specifically, the model achieved a sensitivity of 0.700 (95% CI: 0.499–0.901), a specificity of 0.967 (95% CI: 0.902–1.000), a positive predictive value (PPV) of 0.933 (95% CI: 0.807–1.000), and a negative predictive value (NPV) of 0.829 (95% CI: 0.704–0.953). The overall accuracy was 0.860 (95% CI: 0.764–0.956) (**Supplementary Figure 5**).

The calibration curve showed good agreement between the nomogram-predicted probability of CLNM and the actual observed outcome (**Supplementary Figure 7**). The evaluation metrics of all nomograms revealed that the different nomogram provided different benefit across a wide range of scenarios compared to the “treat all” or “treat none” strategies, confirming its clinical utility (**Tables 4**, **5**).

**Table 4:** Performances comparisons among different approaches measured by Sensitivity, Specificity, PPV, NPV, Accuracy and F-Score based on training cohort.

Model	Sensitivity (95% *CI)*	Specificity (95% *CI)*	PPV (95% *CI)*	NPV (95% *CI)*	Accuracy (95% *CI)*
Clinical+ Intratumor	0.857 (0.799,0.915)	0.914 (0.878,0.950)	0.857 (0.799,0.915)	0.914 (0.878,0.950)	0.893 (0.861,0.924)
Clinical+ intra&Peritumoral	0.874 (0.818,0.930)	0.908 (0.871,0.944)	0.843 (0.783,0.903)	0.927 (0.894,0.96)	0.895 (0.864,0.926)
Clinical	0.760 (0.686,0.833)	0.828 (0.781,0.875)	0.700 (0.624,0.776)	0.867 (0.823,0.911)	0.804 (0.764,0.845)

**Table 5:** Performances comparisons among different approaches measured by Sensitivity, Specificity, PPV, NPV, Accuracy and F-Score based on validation cohort.

Model	Sensitivity (95% *CI)*	Specificity (95% *CI)*	PPV (95% *CI)*	NPV (95% *CI)*	Accuracy (95% *CI)*
Clinical+ Intratumor	0.731 (0.625,0.837)	0.883 (0.818,0.948)	0.817 (0.719,0.915)	0.822 (0.747,0.896)	0.820 (0.761,0.879)
Clinical+ intra&Peritumoral	0.754 (0.649,0.859)	0.885 (0.822,0.949)	0.817( 0.719,0.915)	0.842 (0.770,0.913)	0.832 (0.775,0.89)
Clinical	0.721 (0.609,0.834)	0.840 (0.768,0.912)	0.733 (0.621,0.845)	0.832 (0.759,0.905)	0.795 (0.733,0.857)

### Decision curve analysis of the *CLNM* risk nomogram prediction model

**Figure 6:**
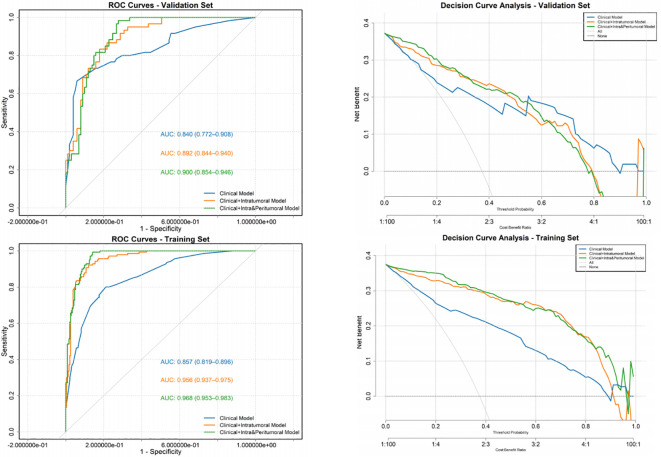
Training and internal validation of the combined clinical and intra-/peritumoral radiomics nomogram: AUC and decision curve analysis.

**Figure 6** show three the area under the curves and decision curve analysis (DCA) curves applied to the *CLNM* risk nomogram prediction model for the training and testing datasets respectively. The DCA curves for both datasets indicate that the model provides substantial standard clinical net benefit and retains stable clinical utility advantages within the threshold probability range of 0.2–0.8. These results suggest the model has practical utility for clinical decision-making in CLNM risk management.

## Discussion

The preoperative identification of central lymph node metastasis (CLNM) in papillary thyroid microcarcinoma (PTMC) represents a significant clinical challenge. While PTMC is defined by a tumor diameter of ≤10 mm and often follows an indolent clinical course, early CLNM is found in up to 60.9% of patients in certain surgical cohorts, complicating the choice between active surveillance (AS) and immediate surgery. According to the American Thyroid Association (ATA) guideline, therapeutic lymph node dissection (LND) is recommended for patients with clinical or imaging evidence of cervical LNM (24). However, unnecessary prophylactic LND carries the risk of complications such as nerve injury, vocal cord palsy, and hypoparathyroidism (24, 25). making accurate preoperative evaluation of paramount significance. To address this limitation, our study introduces a novel, multi-planar radiomics approach designed to transform subjective ultrasound assessment into an objective, quantifiable predictive tool.

A key innovation of this research lies in its dedicated focus on PTMCs and its pioneering use of combined transverse and longitudinal plane analysis for radiomics feature extraction. Traditional ultrasound assessments, such as evaluating the “taller-than-wide” shape, are inherently limited by subjective visual interpretation (1), while CT-based radiomics studies (26–28) may overlook these micro lesions. By systematically extracting high-dimensional features from both planes and incorporating the calculated length-to-width ratio, we have effectively translated vague morphological descriptions into a robust data-driven framework, significantly mitigating inter-observer variability. Our developed nomograms demonstrated exceptional performance, with the clinical model achieving an AUC of 0.840, and the combined Clinical+Intra&Peritumor model maintaining a robust AUC of 0.900. The models were further characterized by excellent calibration and, as decisively confirmed by Decision Curve Analysis (DCA), substantial clinical utility across a wide range of risk thresholds.

Beyond high accuracy, our study introduces the novel concept of configurable models for flexible clinical application. We found that the “Clinical + Intratumoral” model exhibited exceptionally high specificity (0.914), making it ideal for managing low-risk patients inclined toward active surveillance, where avoiding false positives is critical. Conversely, the “Clinical + Peritumoral” model showed the highest sensitivity (0.857), suiting it for high-risk scenarios where minimizing missed diagnoses is paramount. This flexibility allows for truly personalized risk assessment. Lastly, to facilitate clinical implementation, we propose a streamlined workflow where clinicians first perform standard 2D ultrasound to evaluate conventional risk factors like capsule invasion and peritumoral echogenicity, subsequently compute a Rad-score via our platform, and finally integrate these into a nomogram to generate a personalized risk probability categorized into three actionable tiers mapped via SHAP dependence analysis: a low-risk status (probability < 0.20; Rad-score < -0.5) supports the consideration of Active Surveillance (AS) or a more conservative surgical approach to reduce unnecessary intervention, an intermediate-risk status (0.20–0.60; Rad-score -0.5 to 3.5) represents a gray zone necessitating multidisciplinary discussion and closer follow-up, and a high-risk status (probability > 0.60; Rad-score > 3.5) justifies a strong recommendation for prophylactic central neck dissection (PCND) due to the significant likelihood of metastasis.

By extracting quantitative texture features from the 2mm peritumoral expansion zone, we successfully transformed the subjective visual echogenicity change into objective, standardized, and reproducible continuous numerical features. Peritumoral hyperechogenicity emerged as the strongest predictor, likely mirroring pathological alterations in the tumor microenvironment (29, 30). Our analysis further refined this concept, demonstrating that that radiomics serves as a “digital biopsy” of the peritumoral microenvironment. Furthermore, extensive capsular contact (≥50%) was a powerful predictor, underscoring the capsule’s role as a critical barrier to dissemination.

The radiomics signature itself provides deep pathophysiological insights. The strong predictive value of lower intratumoral sphericity quantitatively validates that irregular shape is a hallmark of invasive growth. More importantly, the elevated entropy and heterogeneity in the peritumoral 2-mm region serve as a radiomic surrogate for a disrupted microenvironment, indicative of desmoplastic reaction or early microscopic invasion (31). This aligns with the “seed and soil” hypothesis, where our model simultaneously assesses the intrinsic dysmorphism of the tumor “seed” and the reactive disorder of the peritumoral “soil” (32). By integrating these complementary perspectives, our approach provides a more holistic and powerful assessment of metastatic risk than models based on either region alone.

In conclusion, this study presents a significant advancement in the preoperative management of PTMC. We have developed and validated a novel, multi-planar radiomics-based tool that not only achieves high predictive accuracy for CLNM but also offers unprecedented flexibility for tailored clinical decision-making. By objectively decoding the aggressive phenotypes of both the tumor and its microenvironment, our nomograms empower clinicians to strategically recommend prophylactic central neck dissection for high-risk patients while confidently opting for a more conservative approach in low-risk cases, thereby personalizing surgical care and potentially improving patient outcomes.

### Limitations

This study has several limitations. First, its retrospective and single-center design may introduce selection bias and limits generalizability. Second, we acknowledge the potential for optimism bias inherent in the radiomics studies. Therefore, future prospective studies utilizing diverse, multi-institutional cohorts are essential to confirm the robustness and transportability of our model. Third, while our combined model achieved superior performance, it does not incorporate molecular or genetic markers, such as BRAF V600E mutation, which have been shown to provide additional prognostic information in thyroid cancer.

## Conclusion

We have developed a robust and interpretable nomogram that effectively predicts the preoperative risk of CLNM in PTMC patients. By integrating key sonographic and clinical features, this tool facilitates personalized surgical management, helping to optimize outcomes by tailoring the extent of surgery to individual risk profiles.

## Data Availability

The original contributions presented in the study are included in the article/**Supplementary Material**. Further inquiries can be directed to the corresponding author.
