# Supplementary material for: Predicting central lymph node metastasis in papillary thyroid microcarcinoma: a study of ultrasound and clinical features
Source: Front Endocrinol (Lausanne). 2026 Apr 10;17:1752405. doi: 10.3389/fendo.2026.1752405 (PMC13106017; doi:10.3389/fendo.2026.1752405)
Supplement: Supplementary file 1 [file DataSheet1.docx]

**Supplementary materials**

**Supplementary Table 1**. Quantitative comparison between the present model and key prior predictive models for CLNM in PTMC.

| **Study (Year)** | **Sample Size (Training/Validation)** | **Model Predictors** | **Validation AUC (95% CI)** |
| --- | --- | --- | --- |
| Zhang et al. (2025)  PMID:40771386 | 590/254 | Age, Male sex, Size, Focal infection, Location, Calcification, (Isthmic PTMC focused) | 0.726 |
| Wang et al. (2025)  PMID:41341135 | Meta-analysis (n=15 studies) | Various Clinical + US features | 0.794  (Pooled AUC) |
| Chen et al. (2025)  PMID:40761820 | 263/66 | Age, Diameter, ACR-score,  Rad-score | 0.937 |
| Shi et al. (2026)  PMID:41660496 | 112/48 | Age, Tumor size, Multifocality, and Calcification | 0.811 |
| Zhang et al. (2025)  PMID**:**39865963 | 201/87 | Enhanced US features, Clinical US features, Rad-score | 0.920 |
| Yang et al. (2025)  PMID: 41209233 | 534/230 | Extrathyroidal extension, Multifocality, Macrocalcifications, Clustered microcalcification, Hypo-enhancement, and the elasticity maximum value | 0.78 |
| Tong et al. (2022)  PMID: 35501717 | 300/143 | Radiomics, US-reported lateral LN status | 0.881&0.903  (External validation) |
| Our Study | 373/161 | Clinical + Intra- & Peritumoral Radiomics | 0.900  (0.854–0.946) |

**Radiomic score=-1.2166-1.0962*f₁+0.4604*f_2_-1.5469*f_3_+0.5546*f_4_+0.8257*f_5_-1.1287*f_6_+0.4148*f_7_-0.4584* f_8_**

Rad-score = β₀ + β₁f₁ + β₂f₂ + … + βₙfₙ, where β₀ represents the intercept, and β₁...βₙ denote the weighted coefficients for the selected radiomics features f₁...fₙ.

Where f₁= original_shape2D_Sphericity_Longitudinal.section,

f_2_= lbp.2D_firstorder_InterquartileRange_Cross.section,

f_3_= original_shape2D_Sphericity_Cross.section,

f_4_= lbp.2D_glrlm_RunEntropy_Longitudinal.section2,

f_5_= lbp.2D_gldm_DependenceEntropy_Cross.section2,

f_6_= lbp.2D_glrlm_RunLengthNonUniformityNormalized_Cross.section

f_7_= lbp.2D_firstorder_10Percentile_Longitudinal.section2

f_8_=lbp.2D_gldm_DependenceVariance_Cross.section2

**Probability Conversion Formula:**

P = 1 / (1 + exp(-(Radiomics Score)))

**Supplementary Figure 1. Study Population Inclusion and Data spilt**


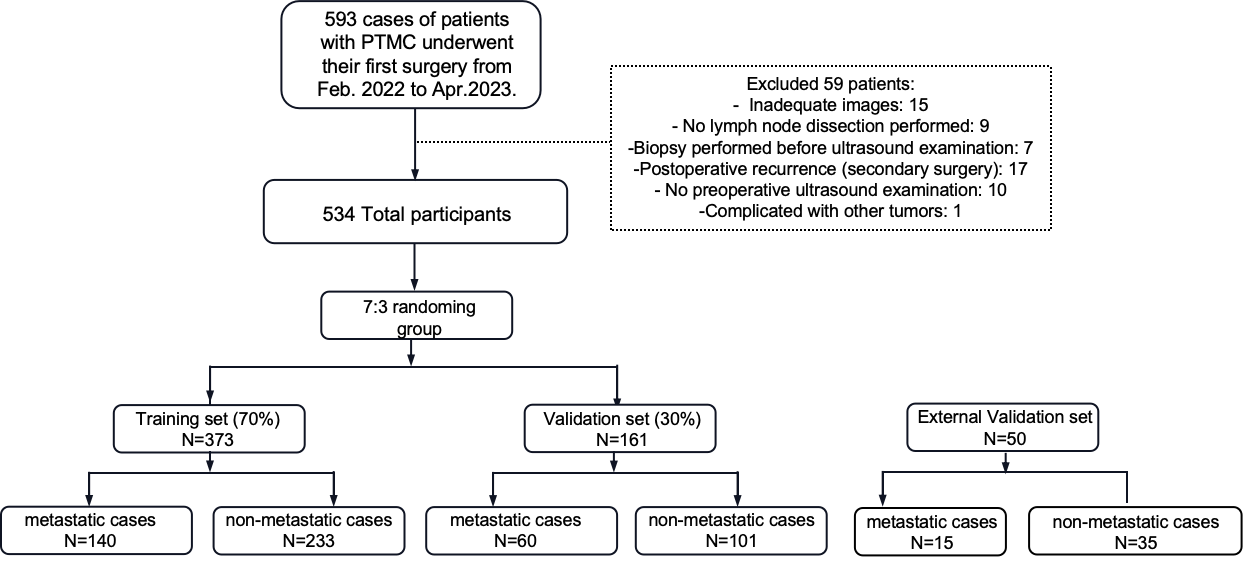


**Supplementary Figure** **2**. SHapley Additive exPlanations dependence plot illustrates the nonlinear contribution of Rad-score to CLNM prediction. Feature importance ranking demonstrates Rad-score as the most consistent predictor across training and validation cohorts, followed by capsule contact and age. Abbreviations: SHAP, SHapley Additive exPlanations; Rad-score, Radiomics score; CLNM,central lymph node metastasis.


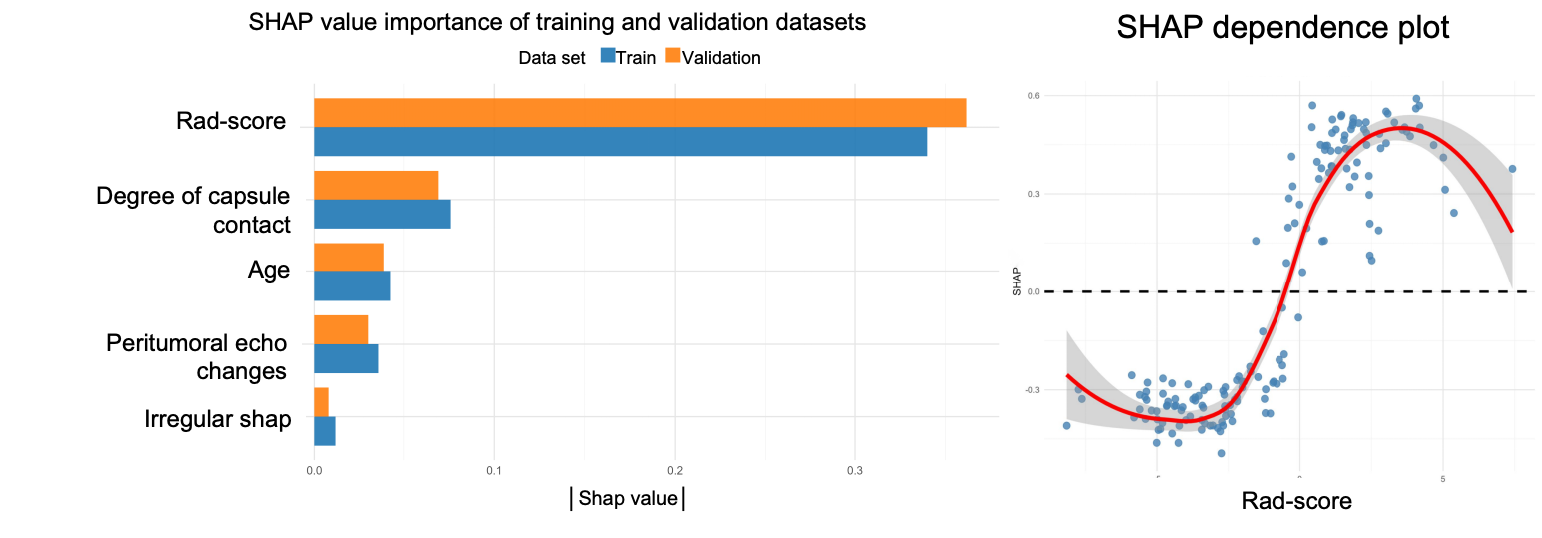


As illustrated in Supplementary Figure 2, the SHapley Additive exPlanations (SHAP) dependence plot reveals the non-linear contribution of the Rad-score to CLNM prediction. Feature importance ranking identified the Rad-score as the most robust predictor across both training and validation cohorts, followed by capsule contact and age.Based on the SHAP dependence analysis for the radiomics score (Rad-score), we identified **key threshold values** that correspond to clinically meaningful risk stratification. The SHAP dependence plot reveals three critical regions:

1. **Zero-contribution point**: Rad-score = -0.5 — where SHAP value crosses from negative to positive, indicating the transition from risk-suppressing to risk-promoting effect.
2. **Sharp increase regions**: Around Rad-score values of -1.2 and 1.4 — where small changes in Rad-score lead to substantial increases in SHAP values, suggesting high sensitivity zones for risk prediction.
3. **Sharp decrease region**: At Rad-score = 3.65 — where further increase in Rad-score paradoxically reduces SHAP values, potentially indicating model saturation or overfitting effects.

Using the pre-defined prediction probability thresholds of 0.20 and 0.60 for risk stratification (Low, Intermediate, High), we mapped these SHAP-identified cut-points to clinically actionable Rad-score thresholds:

| Risk Tier | Prediction Probability | Rad-score Cut-off | SHAP Interpretation |
| --- | --- | --- | --- |
| **Low** | < 0.20 | **< -0.5** | SHAP negative → suppresses predicted probability |
| **Intermediate** | 0.20 – 0.60 | **-0.5 to 3.65** | SHAP positive and increasing → promotes risk |
| **High** | > 0.60 | **> 3.65** | SHAP decreases → possible saturation effect |

Note: The upper bound is rounded from 3.65 to 3.5 for clinical convenience.

**Supplementary Figure 3: Correlation heatmap and Variance Inflation Factor (VIF) analysis of the Clinical + Intratumoral Model and Clinical + Intratumoral and Peritumoral Model**


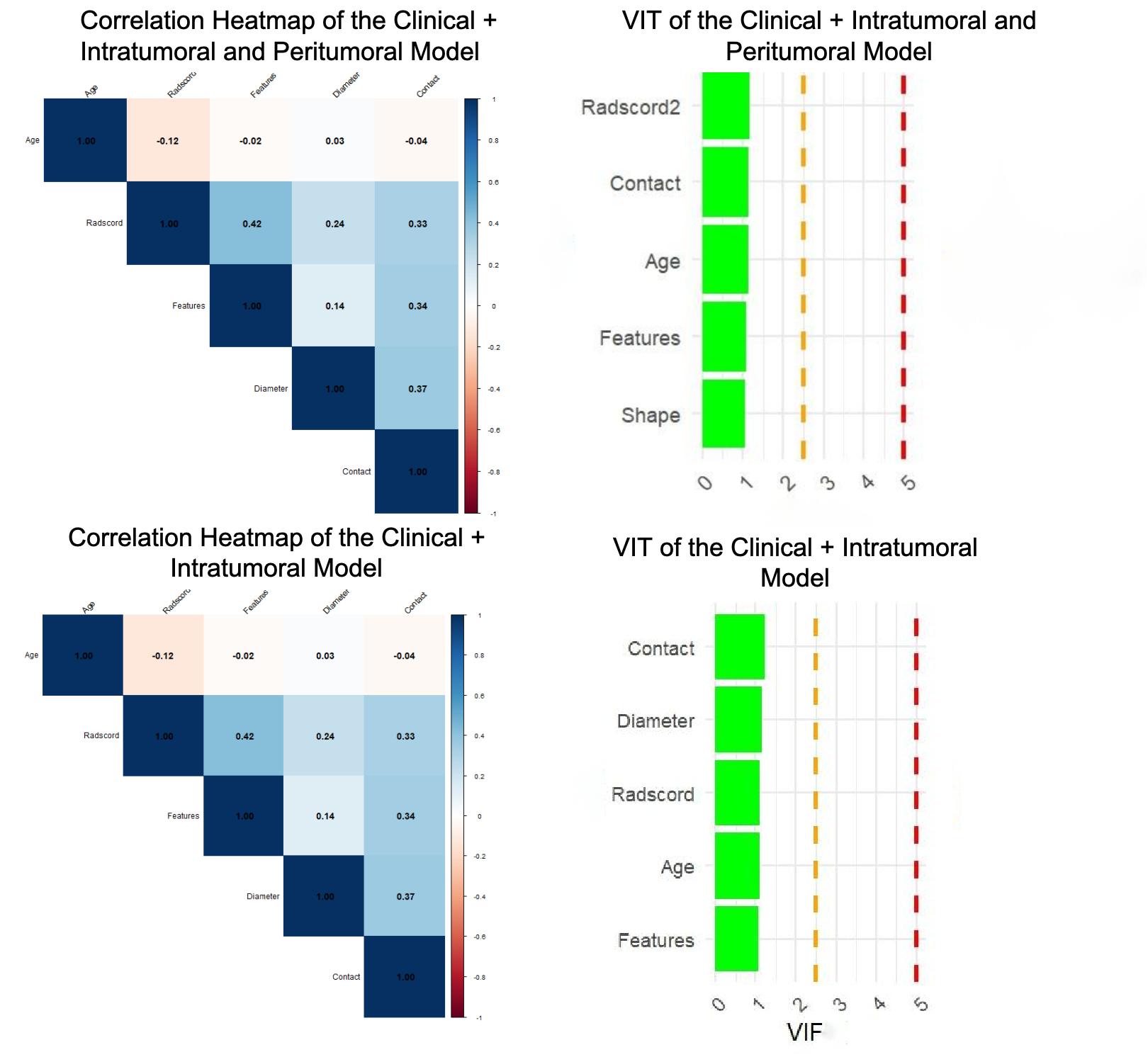


A correlation heatmap visually represents the interrelationships between variables within a dataset in a matrix format. Each cell in the matrix corresponds to the correlation coefficient between two variables, with color intensity intuitively indicating the strength of the correlation. The Pearson correlation coefficient is commonly used for this purpose, ranging from -1 to 1, where -1 denotes a perfect negative correlation, 1 signifies a perfect positive correlation, and 0 indicates no linear correlation.

This quantitative test confirmed the absence of significant multicollinearity among the predictor variables, with all VIF values remaining within an acceptable low range (<5).

**Supplementary Figure 4.** Forest Plot of Intratumoral and Peritumoral Radiomics Features Associated with Central Lymph Node Metastasis in Papillary Thyroid Microcarcinoma

**
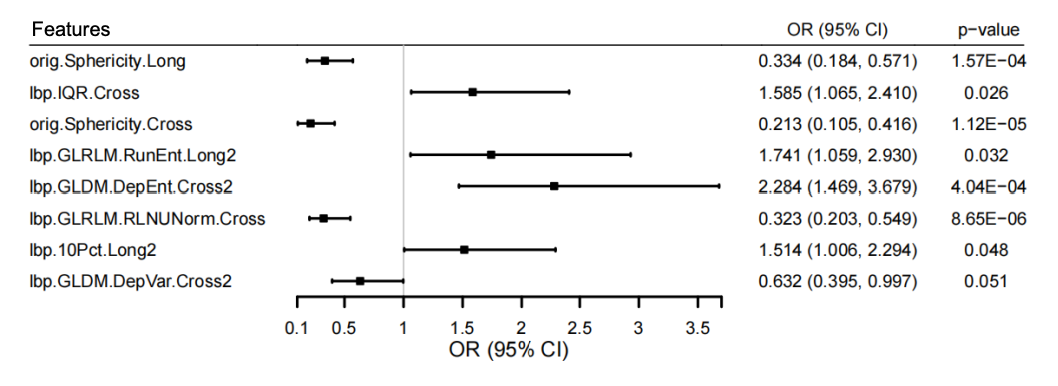
**

Key features included intratumoral shape irregularity (e.g., lower sphericity, OR = 0.21–0.33) and heterogeneity (OR = 1.58), alongside peritumoral textural disorganization (e.g., dependence entropy, OR = 2.28).

**Supplementary Figure 5.** External validation of the combined clinical and intra-/peritumoral radiomics nomogram: AUC and decision curve analysis.


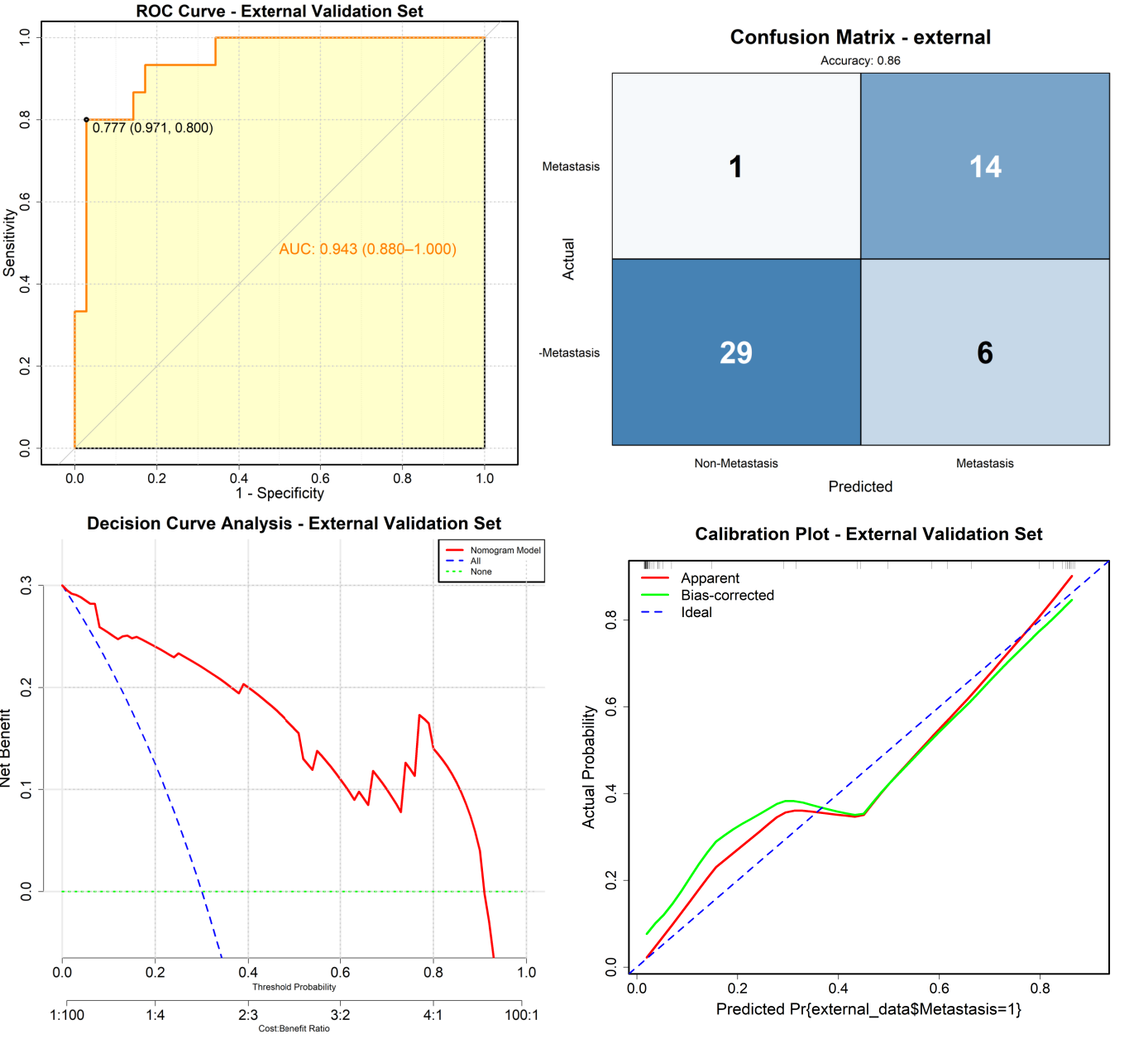


**Table 2. Performances comparisons among Clinical+ intra&Peritumoral approachemeasured by Sensitivity, Specificity, PPV, NPV, Accuracy and F-Score based on external validation cohort.**

| **Model** | **Sensitivity**  **(**95% *CI* **)** | **Specificity**  **(**95% *CI* **)** | **PPV**  **(**95% *CI* **)** | **NPV**  **(**95% *CI* **)** | **Accuracy**  **(**95% *CI* **)** |
| --- | --- | --- | --- | --- | --- |
| Clinical+ intra&Peritumoral | 0.700  0.499, 0.901 | 0.967  (0.902, 1) | 0.933  (0.807, 1) | 0.829  (0.704,0.953) | 0.860  (0.764,0.956) |

**Supplementary Figure 6.** The effectiveness of ComBat harmonization in removing scanner-specific batch effects is visualized in Supplementary Figure 6 via PCA clustering

**
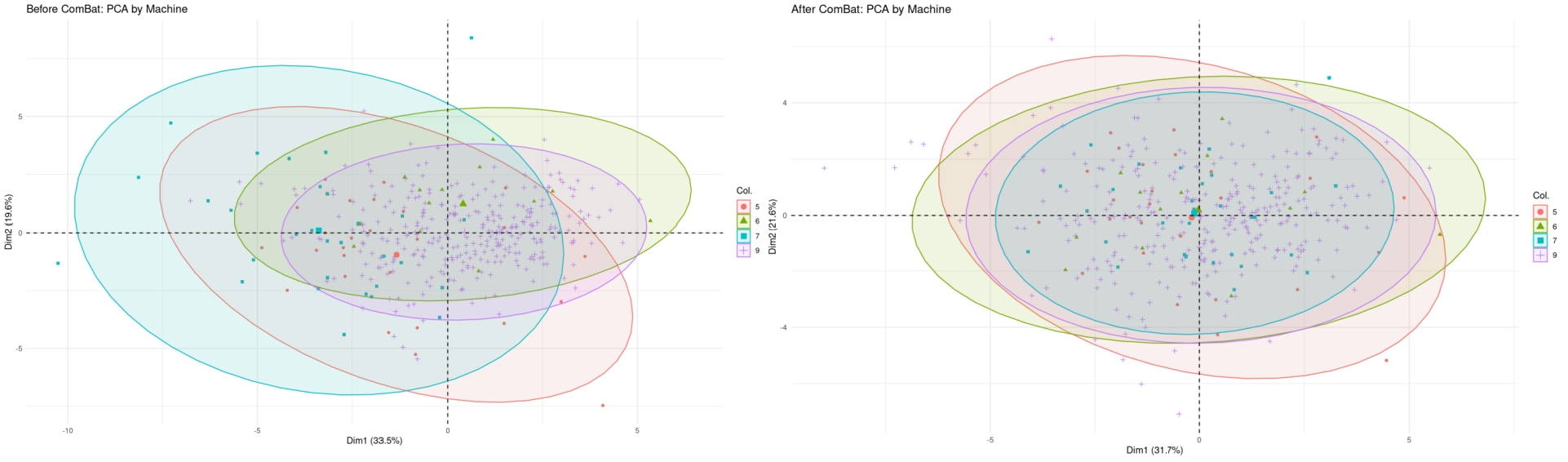
**

**Principal Component Analysis (PCA)** was employed to reduce the dimensionality of the 16 extracted features. Scatter plots of the first two principal components were generated to visualize the global distribution of samples. The presence of batch effects was assessed by observing whether samples clustered according to the imaging device (Machine 5, 6, 7, 9). Confidence ellipses (95% CI) were applied to clusters where sample size permitted to facilitate group comparisons.

**The "Machine" factor accounted for a significant proportion of the total feature variance (R^2^ =0.09462, P < 0.05), confirming the presence of batch effects. After Harmonization: The R^2^ value for the "Machine" factor decreased substantially to 0.00077, and the P-value became non-significant (P > 0.05$).**

Permutational Multivariate Analysis of Variance was performed using the adonis2 function in the vegan R package. **Effective harmonization** was evidenced by a substantial reduction in the R^2^ value for the "Machine" factor and a shift from significant to non-significant p-values (> 0.05). All analyses were conducted in R version 4.5.0.

**Supplementary Figure 7.** The calibration curves of the nomogram-predicted probability of CLNM in training, internal validation data sets.

**
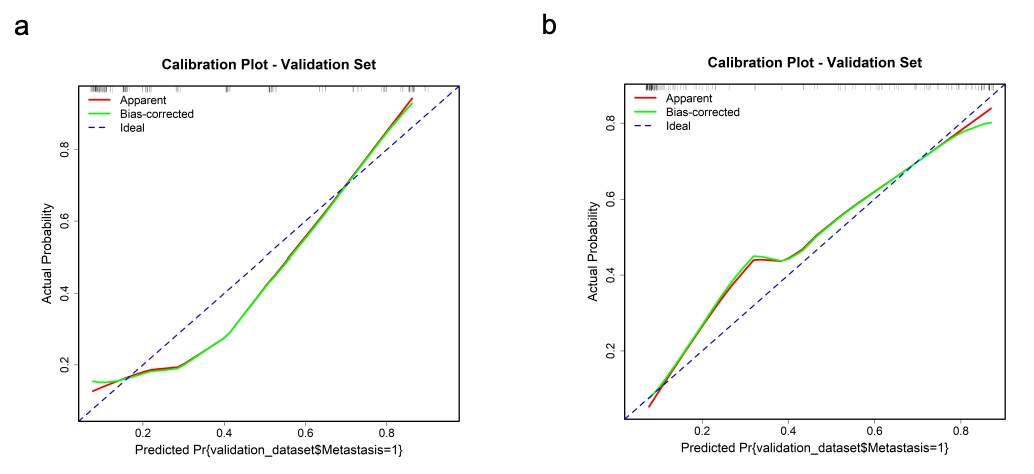
**

a. The calibration curve of training data set, b. The calibration curve of internal validation data set.

**Introduction of transverse and anteroposterior measurement of tumor and ultrasound scan with or without LNM.**

**Statistical Assessment of Batch Effects and Data Harmonization**

**
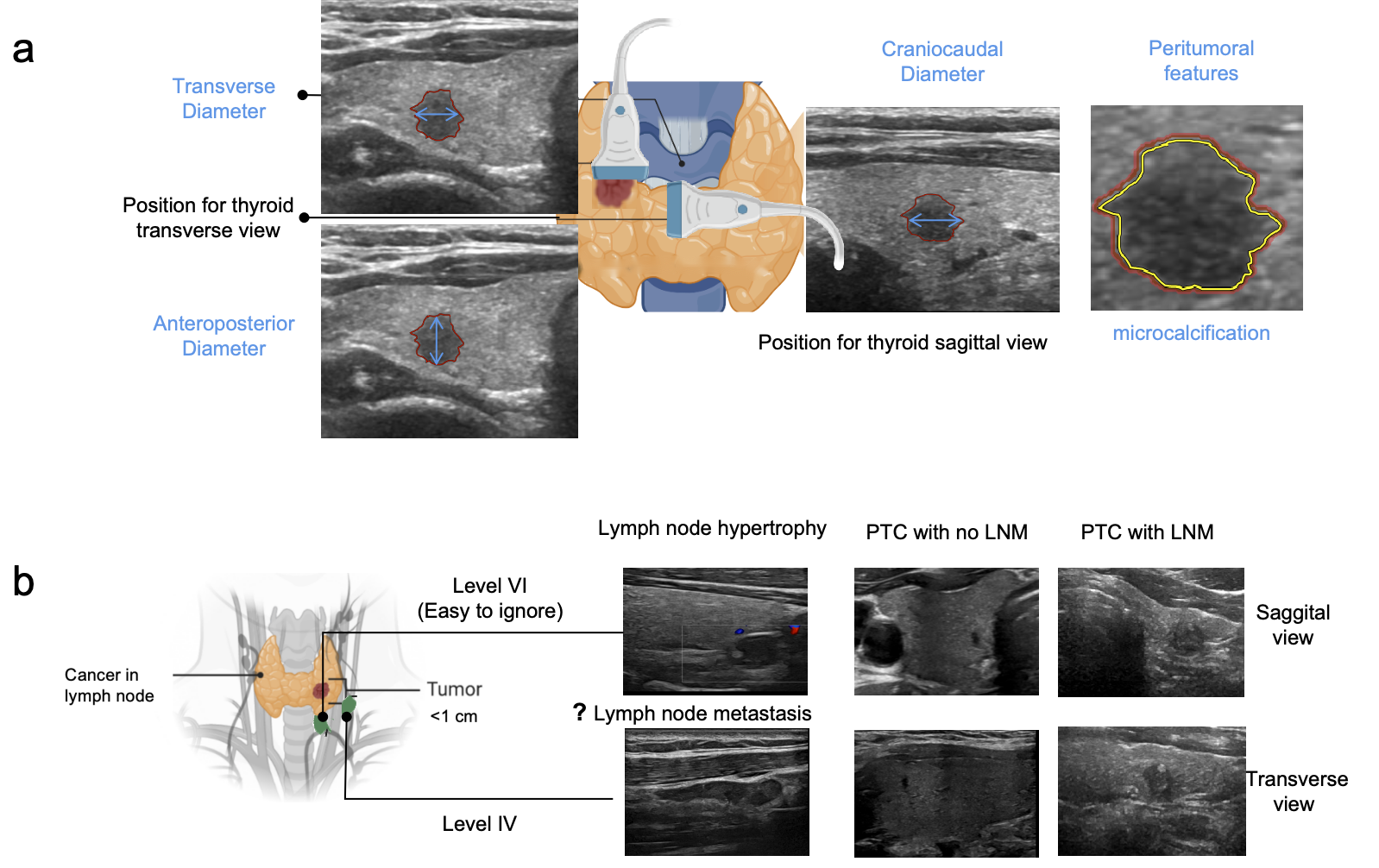
**

**Binary Classification of Thyroid Nodule Features**

All the following characteristics are classified into one of two categories (binary classification) in clinical analysis.

**Location**: Classified as Isthmus or Lobe. Determination is based on the nodule's centroid coordinates relative to the manually segmented thyroid gland mask.

**Shape**: Categorized as Irregular or Regula. Quantified using three features: convexity-to-tumor ratio, compactness, and rectangle-fitting factor.

**Margin**: Classified as Ill-defined or Well-defined. Characterized by eight quantitative features: spiculation, extreme point number, lobule number, moment difference, edge roughness, acutance, local window mean.

**Aspect Ratio**: Categorized as Taller-than-wide or Wider-than-tall. Quantified using three features: elliptical-normalized eccentricity, elliptical-normalized angle, and length-to-width ratio.

**Degree of Capsular Contact**: Classified as≥50% or <50%. Calculated as the ratio of the contact arc length to the total nodule perimeter.

**Homogeneity:** The internal echotexture is classified as Heterogeneous or Homogeneous. Assessed using texture complexity features, including Gray-Level Co-occurrence Matrix (GLCM), Gray-Level Run-Length Matrix (GLRLM), and Neighborhood Gray-Tone Difference Matrix (NGTDM).

**Peritumoral Echogenic Changes**: Determined as Present or Absent. Evaluated by analyzing textural differences in an annular region immediately outside the nodule boundary.

**Posterior Acoustic Pattern:** Classified as Attenuated versus Enhanced/Mixed/Shadowing (grouped as the non-attenuated class). Characterized by analyzing the intensity distribution and texture in the region directly posterior to the nodule.

**Microcalcifications:** Determined as Present or Absent. Detected using feature descriptors sensitive to small, bright, punctate echoes, such as specific filters and high-frequency components of the GLRLM.
